# The Impact of Advertisement Messaging on Enrollment of Young Men Who Have Sex With Men for Web-Based Research: Observational Study

**DOI:** 10.2196/16027

**Published:** 2020-01-13

**Authors:** Holly B Fontenot, Nadia N Abuelezam, Joshua G Rosenberger, David Novak, Kenneth H Mayer, Gregory Zimet

**Affiliations:** 1 Connell School of Nursing Boston College Chestnut Hill, MA United States; 2 The Fenway Institute Boston, MA United States; 3 Department of Biobehavioral Health Penn State University University Park, PA United States; 4 DSN Consulting, LLC Quincy, MA United States; 5 Department of Medicine Beth Israel Deaconess Medical Center Boston, MA United States; 6 Harvard Medical School Boston, MA United States; 7 Section of Adolescent Medicine Indiana University School of Medicine Indianapolis, IN United States

**Keywords:** advertisement, men, sexual minorities

## Abstract

**Background:**

Recruiting young men who have sex with men (YMSM) in community settings is difficult. The use of Web-based social networks and dating apps for recruitment can be successful approaches, although little work has been done on the impact of study advertisement content on recruitment.

**Objective:**

The aim of this study was to evaluate the effects of advertisement message content on the recruitment of YMSM (aged 18-26 years) for a Web-based focus group study, examining perspectives and preferences for a mobile app that was designed to support sexual health among YMSM.

**Methods:**

Between March and April 2017, a recruitment campaign to promote human papillomavirus vaccination was launched on a popular social networking and dating app for YMSM, with 3 different text-based advertisement themes (technology, cancer prevention, and sexual innuendo). The campaign recruited YMSM across 3 states (Massachusetts, New York, and Pennsylvania). We examined the click-through rates, conversion rates, and enrollment rates of each of the advertisements and examined differences in views and clicks by age, state, and time of day.

**Results:**

The sexual innuendo advertisement had the highest click rates when compared with both the technology (click rate ratio [CRR] 2.06, 95% CI 1.74-2.45) and cancer prevention (CRR 1.62, 95% CI 1.38-1.90) advertisements. The sexual innuendo advertisement also had higher study enrollment rates compared with the technology (CRR 1.90, 95% CI 1.23-2.83) and cancer prevention (CRR 2.06, 95% CI 1.37-3.13) advertisements. No differences were observed in clicks or enrollment by age, state, or time of day.

**Conclusions:**

Our marketing campaign, targeting YMSM, was effective in recruiting participants for a qualitative study, using Web-based focus groups. The sexual innuendo advertisement was the most effective and cost-efficient advertisement of the 3 approaches trialed. Different populations need different targeted strategies for study recruitment. Researchers should work with key representatives to develop and test culturally relevant messaging and approaches that utilize current and popular technologies.

## Introduction

### Background

Improving health outcomes among young men who have sex with men (YMSM) is an important goal in the United States [1]. Numerous health disparities exist for this population [2], and health researchers are responding by actively recruiting YMSM into studies to build relevant and culturally appropriate interventions. One of the main difficulties in building relevant and culturally appropriate interventions is recruiting YMSM into feasibility studies. Issues that the youth identify as potential barriers to study enrollment include consent or assent requirements, privacy and confidentiality concerns, time, and scheduling issues [3,4]. Challenges specific to recruitment of YMSM may also include finding places to recruit sexual minorities, increasing privacy and confidentiality concerns related to minority status, and building trust, as well as a history of stigma and discrimination [5]. Recently, investigators have had success in recruiting adolescents/young adults by using Web-based approaches [6-8].

YMSM utilize Web-based resources via mobile devices or personal computers to seek health information and engage socially and sexually with other sexual minority peers [7,9]. Mobile apps for Web-based dating and sexual networks have grown in popularity over the past decade [10]. The popularity of these apps reflects YMSM’s desire to have control over who they interact with and easily find sexual partners [11]. Study recruitment has been done from Web-based social networking apps and websites (eg, Facebook) [6,12] and from dating apps (eg, Grindr and Jack’d) [7,13-15]. Regardless of the Web-based venue in which recruitment is taking place, advertisements are used to attract the attention of prospective study participants. Although some work has been done to understand how the content of imagery on recruitment advertisements influences young men’s engagement in research [6], little work has been done to understand how the thematic messaging of advertisements (in the absence of images) may influence engagement.

### Objectives

During recruitment for a study focusing on human papillomavirus (HPV) vaccination [7], we aimed to understand how different advertisement messaging themes, delivered in a pop-up format on a popular mobile social networking and dating app for YMSM, influenced engagement with those advertisements and ultimate enrollment in the study procedures.

## Methods

### Human Papillomavirus Study Overview

The aim of the larger study was to elicit YMSM’s perspectives and preferences for an app being developed to provide information on HPV, HPV vaccination, and sexual health, as well as referral information and linkages to care. The goal was to enroll 60 YMSM across 3 northeastern states (Massachusetts, New York, and Pennsylvania) for 6 Web-based focus groups (20 YMSM per state and 2 Web-based focus groups per state). Eligibility requirements included (1) self-identification as a man who has sex with men, (2) aged 18 to 26 years, and (3) ability to read and understand English. Initial findings have been published [7].

### Advertisements and Recruitment

**Figure 1:**
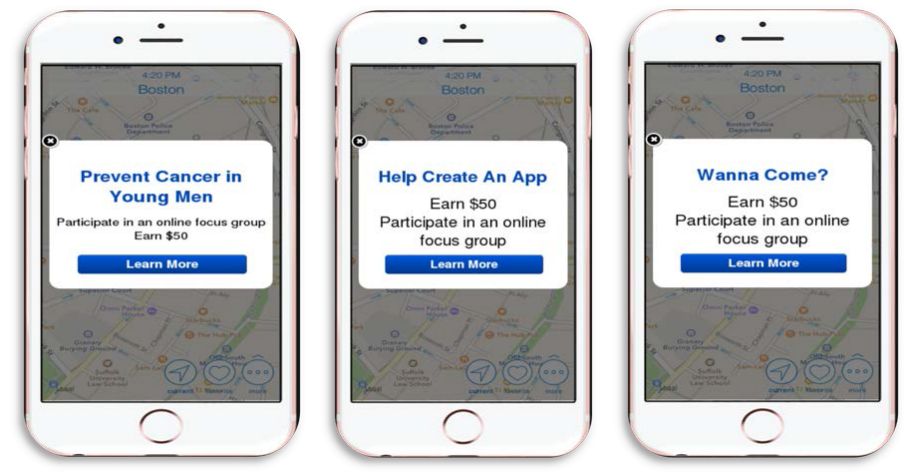
A total of 3 study advertisements with different text-based themes.

Recruitment occurred on a popular global mobile social networking and dating app, utilized by racially diverse YMSM (app name undisclosed per company policy). A total of 3 different pop-up advertisements for study recruitment were created. Pop-up advertisements appeared on the full screen of a mobile device and were compatible for both Android and iOS. Each advertisement included a headline, text (including reference to the US $50 compensation for participation), and a button that would link potential participants to the study website. Each of the 3 advertisements had a different headline that delivered different themes; all the other text was the same for each advertisement (Figure 1). The technology-focused advertisement read as “Help Create an App” and focused on the ultimate technology/app development–related outcome of the pilot study. The cancer prevention–focused advertisement read as “Prevent Cancer in Young Men,” referencing the HPV prevention/vaccination outcome of the larger study. The sexual innuendo–focused advertisement read as “Wanna come?”, referencing an individual’s attendance for Web-based focus groups (with sexual innuendo, as *come* spelled as *cum* is slang for ejaculate/seminal fluid). All advertisements were vetted by key representatives from the population of interest during development.

All advertisements were displayed on the dating app at the same rate during the same recruitment time frame (1 week between March 27 and April 4, 2017) geolocated to 3 different states. Our study marketing campaign comprised 25% of the market shares of all advertisements posted to the dating app during that week. The parent study and all advertisements were approved by Fenway Health Institutional Review Board.

### Project Enrollment

Users of the dating app, who clicked on the link provided on the advertisement, were routed to the study Web page. This Web page included information about the Web-based focus group study, an eligibility survey, and a Web-based consent form. Men who completed the eligibility survey and were eligible to participate were provided with electronic consent forms and were asked to indicate their availability to participate in a 1-hour Web-based focus group. Availability was assessed with a question that provided 3 different dates and times on a drop-down list (the answer choice allowed multiple selections). Dates and times differed depending on the state in which the participant was residing. Participants were given 3 options for scheduling; thereafter, study investigators chose the 2 dates that worked best for the majority of eligible participants. Participants were then emailed invitations to participate for a scheduled focus group.

### Measures

Measures included number of unique views for each advertisement, unique clicks for each advertisement, eligibility survey initiations, eligibility survey completions, men eligible, and men who provided informed consent. From these quantities, unique click-through rates were calculated, representing the number of unique clicks per 100 person views of the advertisement. Conversion rate, representing the number of completions of the eligibility survey per 100 person views of the advertisement and enrollment rate, representing the number of men found eligible and providing consent per 100 person views, were also calculated. In addition, using information on the costs for each of the ads, a calculated cost per enrollment, representing the total cost for the advertisement per enrolled (eligible and consented) participant, was captured.

### Data Analysis

Descriptive statistics for each of the advertisement messages, using information on the state where the advertisement was displayed, and the age of survey takers were calculated. Chi-square tests were used to compare men from different states and different age categories across advertisement types. Rate ratios were calculated (unique click-through rate, conversion rate, and enrollment rate), along with their 95% confidence intervals using conditional maximum likelihood estimates from contingency tables, using the technology-focused advertisement as the reference for the analyses. Analyses were conducted using SAS software version 9.4 (SAS Institute Inc).

## Results

### Overview

**Figure 2:**
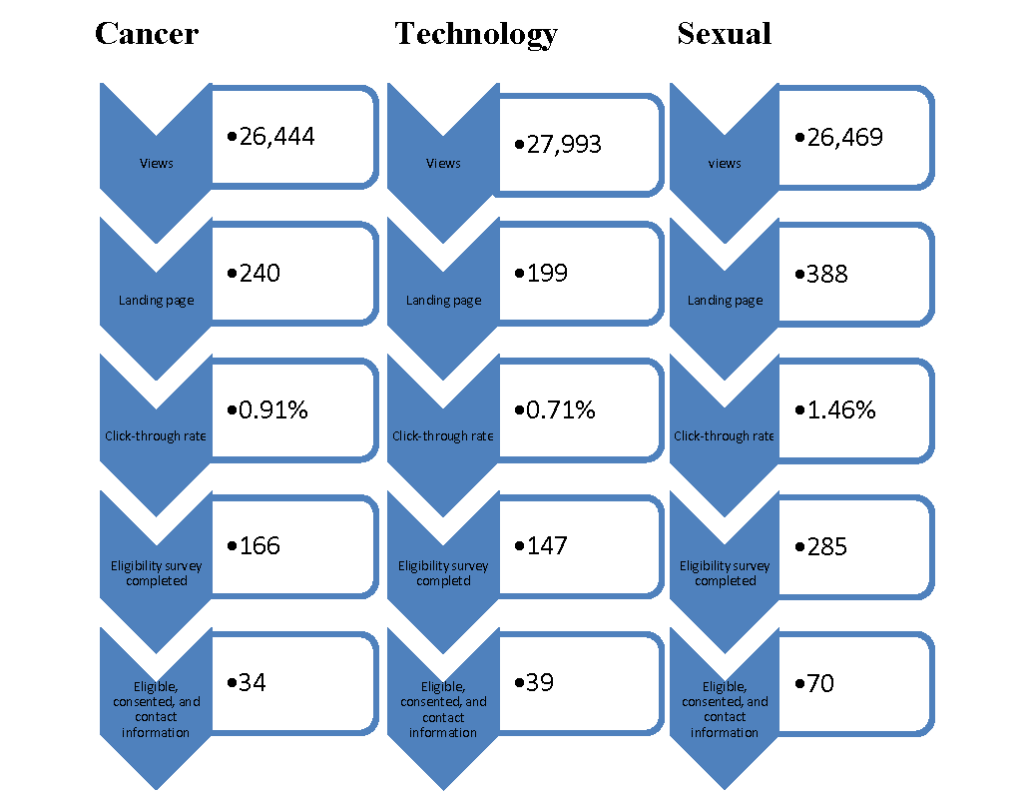
Sexual innuendo advertisement outperformed other advertisements.

Across all advertisements, the campaign reached 80,906 potential participants. The technology advertisement received 27,993/80,906 (34.59%) unique views, the cancer advertisement received 26,444/80,906 (32.68%) unique views, and the sexual innuendo advertisement received 26,469/80,906 (32.71%) unique views (Figure 2). There were 827 unique clicks on the advertisements, resulting in an overall unique click rate of 1.02% (827/80,906). Of those who clicked on the advertisements, 598 participants completed the eligibility survey, resulting in an overall conversion rate of 0.74% (598/80,906). A total of 143 men were eligible for the study, and they provided informed consent, resulting in an overall enrollment rate of 0.18% (143/80,906).

### Advertisement-Specific Results

The theme of the advertisement-affected response rates (Table 1). Click rates were higher for the advertisements that included a sexual innuendo theme (click rate ratio [CRR] 2.06, 95% CI 1.74-2.45) or a cancer theme (CRR 1.28, 95% CI 1.06-1.54) than for the advertisement that had a technology theme (Table 1).

The advertisement that included a sexual innuendo theme also had a higher conversion rate (CRR 2.10, 95% CI 1.73-2.56) and a higher enrollment rate (CRR 1.90, 95% CI 1.23-2.83) than the advertisement that had the technology theme. Cost per enrollment was lowest for the sexual innuendo advertisement at US $21.43 per enrollment and most expensive for the technology advertisement at US $51.72 per enrollment.

### Participants’ Characteristics

Age, state, and time of day the advertisement was viewed were collected for men who completed the eligibility survey after clicking through the advertisement (N=598; Table 2).

More than 50% of all men were in the eligible age range for the study (18-26 years of age). The majority responded from New York (339/598, 56.7%) followed by Pennsylvania (152/598, 25.4%) and Massachusetts (107/598, 17.9%). More men in New York responded to the technology advertisement than in Pennsylvania (27.7% [94/339] vs 20.4% [31/152]), whereas more men in Massachusetts responded to the sexual innuendo advertisement than men in New York (54.2% [58/107] vs 44.3% [150/339]), although none of these differences were statistically significant. We found no differences in advertisement selection on the basis of time of day when the advertisements were viewed.

**Table 1:** Unique click-through, conversion, and enrollment rates are presented for each of the advertisement themes. Cost per enrollment is also reported for each advertisement theme.

Advertisement type		Unique click-through rate (clicks per 100 person views)		Conversion rate (to complete eligibility survey)		Enrollment rate (eligible, consented, and contact info)		Cost per enrollment (US $)
		Mean	CRR^a^ (95% CI)	Mean	CRR (95% CI)	Mean	CRR (95% CI)	
**Sexy advertisement versus tech advertisement**								
	Tech	0.71	Reference	0.54	Reference	0.14	Reference	51.72
	Cancer	0.91	1.28 (1.06-1.54)	0.63	1.16 (0.93-1.45)	0.12	0.92 (0.58-1.46)	44.12
	Sexy	1.47	2.06 (1.74-2.45)	1.13	2.10 (1.73-2.56)	0.26	1.90 (1.23-2.83)	21.43
**Sexy advertisement versus cancer advertisement**								
	Cancer	0.91	Reference	0.63	Reference	0.12	Reference	44.12
	Sexy	1.47	1.62 (1.38-1.90)	1.13	1.81 (1.50-2.19)	0.26	2.06 (1.37-3.13)	21.43

^a^CRR: click rate ratio.

**Table 2:** Characteristics (state of residence, age, and time of day of click) for men who completed the eligibility survey by advertisement theme.

Indicator			Advertisement type					Total (N=598)	
			Tech (n=147)		Cancer (n=166)		Sexy (n=285)		
**State**									
	Massachusetts	22 (20.6)		27 (25.2)		58 (54.2)		107 (17.9)	
	New York	94 (27.7)		95 (28.0)		150 (44.3)		339 (56.7)	
	Pennsylvania	31 (20.4)		44 (29.0)		77 (50.7)		152 (25.4)	
**Age (years)**									
	18-26	88 (25.0)		100 (28.4)		88 (25.0)		352 (59.3)	
	27+	58 (24.0)		64 (26.5)		58 (24.0)		242 (40.7)	
**Time of day**									
	AM	71 (23.8)		87 (29.1)		141 (47.2)		299 (50.0)	
	PM	76 (25.4)		79 (26.4)		144 (48.2)		299 (50.0)	

### Recruitment Results

Of the 598 men who responded to the eligibility survey, 143/598 (23.9%) men met all the eligibility criteria and provided informed consent. Of these, 48 YMSM (13 to 19 per state), with a mean age of 23.4 years, participated in the Web-based focus groups that made up the larger study. Of note, this recruitment strategy was successful in enrolling YMSM of diverse racial and ethnic backgrounds; 70% participants reported race as black, 12% white, 4% Asian, 8% more than one race, and 6% other and 22% reported Hispanic ethnicity [7].

## Discussion

### Principal Findings

Our marketing campaign on 1 popular social media app that is oriented to YMSM seeking social and sexual relationships was effective in recruiting for Web-based focus groups. The campaign was rapid (approximately 1 week) and effective (overall conversion rate of 0.74% [598/80,906], leading to 143 eligible and consented individuals), and the enrollment of a racially and ethnic diverse sample of YMSM (n=48) for a qualitative study focused on HPV. The sexual innuendo theme was the most effective text-based advertisement out of the 3 text-based advertisement themes trialed.

Although all advertisements had the same market share and views, the sexual innuendo advertisement outperformed the other advertisements. The sexual innuendo advertisement had, approximately 2 times, the individuals click to the landing page, complete the eligibility survey, and report being eligible, as well as provide consent and contact information for further engagement (Figure 2). This advertisement was also the most cost effective. During the subsequent Web-based focus groups, YMSM noted that fun and sexy advertisements were a facilitator to research participation. Other researchers have also reported successful recruitment of YMSM for HPV- [6,8,12] or HIV-focused [13,16] studies on social media, nationally and internationally. Reiter et al [6] tested image-based advertisements on Facebook and reported a similar overall conversion rate of 0.66% (our study: 0.74%). They reported higher conversion rates for advertisements that had images of a couple, and advertisements with text mentioning sexually transmitted diseases had higher click-through rates as compared with advertisements with text mentioning cancer [6]. Martinez et al [16] found Facebook to also be effective, and these researchers also reported success in recruiting Latino gay couples. Finally, Buckingham et al [13] found recruitment on Grindr (an app very similar to the app used for recruitment in this study) the most effective as compared with recruitment on other social media platforms. These researchers also reported Grindr recruitment to be the most rapid, yielding a large number of potential participants in the shorter period of time [13].

Adolescents and young adults are using websites and apps to engage socially and to seek health information [17,18], and creative marketing campaigns for research is becoming an increasingly important strategy for engagement [19]. Recruitment of YMSM and other marginalized youth for research may be challenging; however, increasingly, success has been noted with recent recruitment strategies on the Web [6,7,13,16].

### Strengths and Limitations

Our results should be considered in terms of our study limitations. Our recruitment strategy (utilizing a dating app) may not be reflective of all YMSM who could benefit from HPV vaccination or who may be interested in research. YMSM who accessed the app generally did so to meet sexual partners, so they might have been more responsive to sexually oriented advertisements. Therefore, the sexual innuendo advertisement was potentially the best advertisement targeted to the audience. In addition, it is possible that YMSM responded positively to this advertisement, as it was uniquely phrased as an invitation or a question, and this phrasing may tap into young adults’ desires to engage in new or perceived riskier (because of the sexual innuendo) opportunities. Future research in different Web-based environments may find different results. We were recruiting for a 1-time/1-hour Web-based focus group. Recruitment for longitudinal studies may be more difficult; however, our findings support that the youth will re-engage to participate in qualitative studies on the Web (Web-based focus groups), after the initial recruitment eligibility survey. We also offered compensation of US $50. Recruitment may be more difficult with lower remuneration, especially for longitudinal studies. In addition, the font size for compensation on the cancer advertisement was slightly smaller than the other 2 advertisements. This potentially could have had an effect on the findings. Another limitation is that this study focused on 3 large northeastern US cities. As a result, the findings and preferences noted here may not apply to recruitment of YMSM participants in small cities or in other geographic locations.

Overall, we were successful in recruiting a diverse sample of YMSM for qualitative research, despite not screening for ethnicity and race in the initial eligibility survey. Our results point to the importance of considering not just the platforms and locations used for recruitment (eg, Facebook, dating apps, and community settings) but also the phrasing and content of recruitment advertising.

### Conclusions

Different populations need different targeted strategies for recruitment, including study advertisement messaging. We were successful with a technology-driven, effective, and rapid approach to recruit a diverse sample of YMSM for a qualitative study, Web-based focus groups. The text-based advertisement with a sexual innuendo theme was most effective. Researchers should collaborate with representatives from their population of interest to develop effective and culturally relevant study recruitment strategies that capitalize on current and popular technologies.

## Abbreviations

CRR: click rate ratio
HPV: human papillomavirus
YMSM: young men who have sex with men

## References

[1] HealthyPeople.

[2] Kann L, McManus T, Harris WA, Shanklin SL, Flint KH, Queen B, Lowry R, Chyen D, Whittle L, Thornton J, Lim C, Bradford D, Yamakawa Y, Leon M, Brener N, Ethier KA (2018). Youth risk behavior surveillance - United States, 2017. MMWR Surveill Summ.

[3] Shaw SM, Caldwell LL, Kleiber DA (1996). Boredom, stress and social control in the daily activities of adolescents. J Leisure Res.

[4] Fredricks JA (2012). Extracurricular participation and academic outcomes: testing the over-scheduling hypothesis. J Youth Adolesc.

[5] Prescott TL, Phillips GI, DuBois LZ, Bull SS, Mustanski B, Ybarra ML (2016). Reaching adolescent gay, bisexual, and queer men online: development and refinement of a national recruitment strategy. J Med Internet Res.

[6] Reiter PL, Katz ML, Bauermeister JA, Shoben AB, Paskett ED, McRee A (2017). Recruiting young gay and bisexual men for a human papillomavirus vaccination intervention through social media: the effects of advertisement content. JMIR Public Health Surveill.

[7] Fontenot HB, Rosenberger JG, McNair KT, Mayer KH, Zimet G (2019). Perspectives and preferences for a mobile health tool designed to facilitate HPV vaccination among young men who have sex with men. Hum Vaccin Immunother.

[8] Gerend MA, Madkins K, Phillips G, Mustanski B (2016). Predictors of human papillomavirus vaccination among young men who have sex with men. Sex Transm Dis.

[9] Sun CJ, Stowers J, Miller C, Bachmann LH, Rhodes SD (2015). Acceptability and feasibility of using established geosocial and sexual networking mobile applications to promote HIV and STD testing among men who have sex with men. AIDS Behav.

[10] Macapagal K, Moskowitz DA, Li DH, Carrión A, Bettin E, Fisher CB, Mustanski B (2018). Hookup app use, sexual behavior, and sexual health among adolescent men who have sex with men in the United States. J Adolesc Health.

[11] Miller B (2015). 'They’re the modern-day gay bar': Exploring the uses and gratifications of social networks for men who have sex with men. Comput Human Behav.

[12] Das R, Machalek DA, Molesworth EG, Garland SM (2017). Using Facebook to recruit young Australian men into a cross-sectional human papillomavirus study. J Med Internet Res.

[13] Buckingham L, Becher J, Voytek CD, Fiore D, Dunbar D, Davis-Vogel A, Metzger DS, Frank I (2017). Going social: success in online recruitment of men who have sex with men for prevention HIV vaccine research. Vaccine.

[14] Burrell ER, Pines HA, Robbie E, Coleman L, Murphy RD, Hess KL, Anton P, Gorbach PM (2012). Use of the location-based social networking application GRINDR as a recruitment tool in rectal microbicide development research. AIDS Behav.

[15] Pines HA, Gorbach PM, Weiss RE, Hess K, Murphy R, Saunders T, Brown J, Anton PA, Cranston RD (2013). Acceptability of potential rectal microbicide delivery systems for HIV prevention: a randomized crossover trial. AIDS Behav.

[16] Martinez O, Wu E, Shultz AZ, Capote J, Rios JL, Sandfort T, Manusov J, Ovejero H, Carballo-Dieguez A, Baray SC, Moya E, Matos JL, DelaCruz JJ, Remien RH, Rhodes SD (2014). Still a hard-to-reach population? Using social media to recruit Latino gay couples for an HIV intervention adaptation study. J Med Internet Res.

[17] Wartella E, Rideout V, Montague H, Beaudoin-Ryan L, Lauricella A (2016). Teens, health and technology: a national survey. Media Commun.

[18] Anderson M, Jiang J (2018). Public Services Alliance.

[19] Ashley C, Tuten T (2015). Creative strategies in social media marketing: an exploratory study of branded social content and consumer engagement. Psychol Mark.

